# Leukocyte transmigration into tissue-engineered constructs is influenced by endothelial cells through Toll-like receptor signaling

**DOI:** 10.1186/scrt533

**Published:** 2014-12-20

**Authors:** Sushma Bartaula-Brevik, Torbjorn O Pedersen, Anna L Blois, Panagiota Papadakou, Anna Finne-Wistrand, Ying Xue, Anne Isine Bolstad, Kamal Mustafa

**Affiliations:** Department of Clinical Dentistry, Center for Clinical Dental Research, University of Bergen, Årstadveien 19, N-5009 Bergen, Norway; Department of Biomedicine, University of Bergen, Jonas Lies vei 91, 5009 Bergen, Norway; Centre for Cancer Biomarkers, Department of Clinical Medicine, Section for Pathology, University of Bergen, Jonas Lies vei 91B, 5021 Bergen, Norway; Children’s Hospital Boston, Vascular Biology Department, Harvard Medical School, 300 Longwood Avenue, Boston, MA USA; Department of Fiber and Polymer Technology, KTH Royal Institute of Technology, Teknikringen 42, SE-100 44 Stockholm, Sweden; Department of Clinical Dentistry - Periodontics, University of Bergen, Årstadveien 19, 5009 Bergen, Norway

## Abstract

**Introduction:**

Inflammation plays a crucial role in tissue regeneration, wound healing, and the success of tissue-engineered constructs. The aim of this study was to investigate the influence of human umbilical vein endothelial cells (ECs) on leukocyte transmigration when co-cultured with primary human bone marrow-derived multipotent stromal cells (MSCs).

**Methods:**

MSCs with and without ECs were cultured in poly (L-lactide-*co*-1, 5-dioxepan-2-one) (poly (LLA-co-DXO)) scaffolds for 1 week *in vitro* in a bioreactor system, after which they were implanted subcutaneously in non-obese diabetic/severe combined immunodeficient mice. After 1 and 3 weeks, scaffolds were retrieved, and the mRNA expression of interleukin 1-beta (IL-1β), IL-6, IL-10, hypoxia-inducible factor 1-alpha (HIF-1α), HIF-1β, and mammalian target of rapamycin was examined by real-time reverse transcription-polymerase chain reaction. Furthermore, immunofluorescent staining was performed for IL-1β, IL-6, neutrophils, and CD11b. In addition, Western blotting was done for IL-1β and IL-6. Leukocyte transmigration genes and genes in Toll-like receptor pathways, expressed by MSCs cultured *in vitro* with or without ECs, were further investigated with a microarray dataset.

**Results:**

*In vitro*, genes involved in leukocyte transmigration and Toll-like receptor pathways were clearly influenced by the addition of ECs. Platelet/endothelial cell adhesion molecule-1 (PECAM-1) and cadherin-5 (CDH5), both genes involved in leukocyte transmigration, were expressed significantly higher in the MSC/EC group.

*In vivo*, the MSC/EC group showed higher mRNA expression of hypoxia-inducible factors HIF-1α and HIF-1β. The mRNA expression of anti-inflammatory cytokine IL-10 showed no significant difference, whereas the mRNA and protein expression of pro-inflammatory cytokines IL-1β and IL-6 were lower in the MSC/EC group. The quantitative analysis of immunofluorescent staining revealed a significant difference in the number of neutrophils migrating into constructs, with the highest density found in the MSC/EC group. The number of macrophages positive for IL-6 and CD11b was significantly reduced in the MSC/EC group.

**Conclusions:**

The recruitment of leukocytes into tissue-engineered constructs with MSCs is strongly influenced by the addition of ECs via activation of leukocyte transmigration and Toll-like receptor pathways.

## Introduction

Re-establishing the function of lost tissues is the ultimate goal of tissue engineering. The process of regenerating complex tissues is, however, not only dependent on progenitor cells differentiating to specialized parenchymal cells. Development of an adequate blood supply is required to ensure survival of implanted cells as well as development and maintenance of the tissue. Under defined mechanochemical culture conditions, primary human bone marrow-derived multipotent stromal cells (MSCs) can differentiate into osteoblasts, chondrocytes, adipocytes, myocytes, and neuronal-like cells [1, 2]. The differentiation of MSCs into endothelial cells (ECs) has been shown [3] but is controversial [4]. Pre-vascularization through co-seeding of MSCs and ECs has therefore been performed to generate tissue-engineered constructs with an intrinsic vasculature upon implantation *in vivo*[5–8].

The influence of ECs on osteogenic differentiation of MSCs has been extensively studied, identifying ECs as an important regulator of MSC commitment to the osteogenic lineage [9–11]. Microarray data have shown that ECs modulate the gene expression profile of MSCs, in particular through the transforming growth factor-beta pathway [12]. In addition, recent work has identified MSCs as appropriate perivascular cells in tissue-engineered constructs containing both ECs and MSCs [13]. The communication between the two cell types is a combination of juxtacrine and paracrine signaling. Vascular assembly has an obvious requirement for direct contact communication, with MSCs regulating EC proliferation, vessel diameter, and maturation of the developing vasculature [6, 7]. However, the release of bioactive molecules (cytokines, chemokines, and growth factors) is a significant part of the cellular cross-talk and alters the signal delivered to surrounding tissues after *in vivo* implantation, thus playing a vital role in the success of the constructs.

Surgical procedures induce acute inflammation that triggers wound healing, repair, and regeneration [14, 15]. Also, implantation of cells and biomaterials is likely to result in a combination of acute and chronic inflammatory stimulation to surrounding tissues. In addition, MSCs have been shown to interact with immune cells and modulate their functional activities through the release of anti-inflammatory cytokines [16, 17].

In some cases, fibrosis hinders vascularization, which leads to a necrotic core of implanted tissue-engineered constructs. Inflammation and angiogenesis are co-dependent processes in certain pathological processes and in wound healing [18]. A certain level of inflammation is therefore favorable for vascular ingrowth and degradation of the scaffold material and subsequently in achieving the maximal level of regeneration and implant success [19, 20].

The vascular endothelium facilitates leukocyte transmigration upon chemotactic signals from damaged or hypoxic tissues. The effects of including a vascular endothelium in a tissue-engineered construct on migration of leukocytes are, however, not well described. We studied the transmigration of leukocytes involved in acute and chronic inflammation into constructs with or without an intrinsic vasculature and the molecular mechanisms behind its modulation.

## Materials and methods

### *In vitro*cultivation of cells

Primary human bone marrow-derived MSCs were purchased from StemCell Technologies (Vancouver, BC, Canada). Purity of the cells was confirmed by flow cytometry, which showed that more than 90% of the cells expressed CD29, CD44, CD105, and CD166 and less than 1% expressed CD14, CD34, and CD45. The MSCs were cultured in MesenCult complete medium (StemCell Technologies) in accordance with the instructions of the manufacturer.

Human umbilical vein ECs were purchased from Lonza (Clonetics®, Walkersville, MD, USA). ECs were expanded in EC growth medium (EGM®) (Lonza) containing 500 mL EC basal medium and supplements: fetal bovine serum 10 mL, bovine brain extract [21] 2 mL, human endothelial growth factor 0.5 mL, hydrocortisone 0.5 mL, and GA-1000 0.5 mL. Cells no older than passage five were used, and all cells were cultured at 37°C and 5% CO_2_.

### Production of scaffolds

Poly(L-lactide-*co*-1,5dioxepan-2-one) [poly(LLA-co-DXO)] scaffolds were prepared as previously described [22, 23]. Briefly, porous scaffolds (pore sizes of 90 to 500 μm) were produced from co-polymer poly(LLA-co-DXO) by a solvent-casting particulate-leaching method. The sterilization of scaffolds was carried out in a pulsed electron accelerator operating at 6.5 MeV (Mikrotron, Acceleratorteknik, The Royal Institute of Technology, Stockholm, Sweden) with a radiation dose of 2.5 Megarad (Mrad) in an inert atmosphere.

### Preparation for *in vivo*implantation

The scaffolds with cells seeded for *in vivo* implantation were prepared in a similar way as previously described [6, 24]. Briefly, scaffolds 12 mm in diameter and 1.5 mm thickness were prewet with MesenCult complete medium (StemCell Technologies) and incubated overnight at 37°C and 5% CO_2_. Then, 5 × 10^5^ cells were seeded per scaffold, either MSCs alone or MSCs/ECs in a 5:1 ratio. To facilitate distribution of cells, an orbital shaker (Eppendorf, Hamburg, Germany) was used, and cells were allowed to attach overnight before scaffolds were transferred to separate modified spinner flasks (Wheaton Science, Millville, NJ, USA) for 1 week in a dynamic culture system with 50 rotations per minute. After 1 week *in vitro*, 6-mm discs were punctured with a dermal skin puncher and the scaffolds were implanted *in vivo.*

### Animal experiments

The animal experiments were performed after approval from the Norwegian Animal Research Authority and conducted according to the European Convention for the Protection of Vertebrates used for Scientific Purposes (local approval number 3029). Non-obese diabetic/severe combined immunodeficient (NOD/SCID) mice (n = 15) purchased from Taconic Farms (Bomholtgård Breeding and Research Center, Ry, Denmark) were used in this study. The animals were kept in sterile polystyrene cages containing wood shavings in a climate-controlled environment with 12 dark/light cycles and fed with standard rodent chow and water *ad libitum*. The animals were 6 to 8 weeks at the time of the surgery. An intramuscular injection of 20 μL of Rompun (Xylazin) (20 mg/mL) (Bayer Health Care, Leverkusen, Germany) and Narketan (Ketamin) (Vétoquinol, Lure, France) in 1:2 ratios was performed to anesthetize the animals. On the back of the mice, a 2.5-cm incision was made providing sufficient space for subcutaneous implantation of scaffolds. A scaffold for each experimental group (MSC or MSC/EC) was placed in the mouse, and six mice were used for each time point. For the 3 weeks of implantation, an additional six empty scaffolds were implanted in three mice. Wounds were closed with Vetbond™ Tissue Adhesive (n-butyl cyanoacrylate) (3M™, St. Paul, MN, USA). After 1 or 3 weeks of implantation, animals were euthanized with deep isoflurane (Schering Plough, Kenilworth, NJ, USA) anesthesia followed by cervical dislocation, after which the implanted scaffolds were carefully dissected and retrieved. The samples were then cut into two halves, and each half was further processed for real-time reverse transcription-polymerase chain reaction (RT-PCR) analysis or histological embedding.

### Real-time reverse transcription-polymerase chain reaction

RNA was extracted from the scaffolds by using an E.Z.N.A. Total RNA Kit (Omega Bio-Tek, Norcross, GA, USA) after being snap-frozen in liquid nitrogen. Quantifications and determination of RNA purity were performed with a NanoDrop Spectrophotometer (ThermoScientific NanoDrop Technologies, Wilmington, DE, USA). A high-capacity cDNA Archive Kit (Applied Biosystems, Carlsbad, CA, USA) was used for the reverse transcription reaction. Total RNA (1,000 ng) was mixed with nuclease-free water, reverse transcriptase buffer, random primers, deoxyribonucleotide triphosphate (dNTP), and MultiScribe reverse transcriptase. Standard enzyme and cycling conditions were applied for 2 minutes at 50°C and 20 seconds at 95°C, followed by 1 second at 95°C and 20 seconds at 60°C per cycle (40 cycles). cDNA corresponding to 10 ng mRNA in each reaction was prepared in duplicates since the standard deviation between the duplicates was minimal for each target gene. Real-time RT-PCR was performed on a StepOnePlus™ real-time PCR system (Applied Biosystems). Mouse-specific TaqMan gene expression assays (Applied Biosystem) were used (Table 1). Data analysis was performed with a comparative threshold cycle (Ct) method with glyceraldehyde 3-phosphate dehydrogenase (GAPDH) as endogenous control [25].

**Table 1:** **List of TaqMan** **probes used for quantitative real-time reverse transcription-polymerase chain reaction analysis**

Gene symbol	Assay ID	Lot number
*GAPDH*	VIC MGB	4352339E
*TNFα*	Mm 00443260_g1	1172346
*IL1β*	Mm 00434228_m1	1172142
*IL6*	Mm 00446190_m1	1169749
*IL4*	Mm 00445259_m1	1173207
*IL10*	Mm 00439614_m1	1172560
*iNOS*	Mm 00440502_m1	1171338
*HIF1α*	Mm 00468869_m1	P121207-003A02
*HIF1β (ARNT)*	Mm 00507836_m1	P110804-009B06
*mTOR*	Mm 0044968_m1	P110708-007D02

### Histological staining

The remaining part of the sample was immediately embedded in optimal cutting temperature compound (Tissue-Tek O.C.T., Sakura Finetek, Tokyo, Japan) and kept at −80°C. Cryosectioning was done with Leica CM 3050S (Leica Microsystems, Wetzlar, Germany) at −24°C with 8-μm-thick sections. Sections from the middle part of the scaffolds were used for immunostaining. Samples intended for paraffin sectioning were fixed in 4% paraformaldehyde. Sections from the middle part of the samples were deparaffinized and stained with hematoxylin and eosin. Sections obtained after 3 weeks of implantation were incubated with primary antibodies in blocking buffer overnight at 4°C and with secondary antibodies for 2 hours the following morning. Nuclei were stained with 4′,6-diamidino-2-phenylindole (DAPI) (1:1,000) for 2 minutes, and the slides were mounted with Prolong® Gold Antifade Reagent (Invitrogen, Carlsbad, CA, USA) before imaging. Exposure time and fluorescence intensities were normalized to appropriate control images.

Rabbit polyclonal anti-mouse interleukin-1-beta (IL-1β) antibody (Abcam, Cambridge, UK), rat monoclonal anti-mouse neutrophil antibody (NIMP) (Abcam), rabbit polyclonal anti-mouse IL-6 antibody (Abcam), and rat monoclonal anti-mouse CD11b antibody (BD Biosciences, San Jose, CA, USA) were used as primary antibodies. Goat anti-rabbit fluorescein isothiocyanate (Santa Cruz Biotechnology, Santa Cruz, CA, USA) and Alexa Fluor 546 goat anti-rat (Invitrogen) were used as secondary antibodies. Double staining with IL-1β and NIMP antibodies was performed to identify the number of IL-1β-positive cells and the number of neutrophils, respectively. Double staining with IL-6 and CD11b antibodies was performed to co-localize macrophages or monocyte-derived cells and their production of IL-6.

### Quantification of immunostaining

On each slide, five sections from one scaffold were mounted. Each section on the slides was divided onto five measuring grids starting from top to bottom in vertical direction. Five sections on each slide (average for the mouse) and five measuring grids in each section (average for the section) were used for the image quantification. The images were taken with Zeiss AxioVision 4.8.1 (Carl Zeiss, Toronto, ON, Canada) at magnification of 10×, and the files were exported as JPEG standard. The measuring grids in each section were noted carefully to avoid any overlap while taking the images. In each section, the areas with no cells were excluded. NIS elements AR 3.2 software (NIS elements, Tokyo, Japan) was used for quantifications. First, the threshold was defined for each channel: red, green, and blue. Next, all the channels were simultaneously referred before counting the cells to avoid misinterpretation. The blue channel was referred continuously to visualize the nuclei, and then the counting was done in the red and green channels separately. Finally, co-localized cells were counted for red and green fluorescent staining together. The number of counted cells per measuring grid was exported to a Microsoft Excel (Microsoft Corporation, Redmond, WA, USA) file before statistical analysis was performed.

### Western blotting

Protein extraction was performed in accordance with the protocol of Chomczynski [26]. Briefly, protein precipitation from the organic phase was prepared by adding isopropanol. Precipitate was washed with ethanol and dissolved in 0.5% sodium dodecyl sulfate solution. Quantifications and determination of protein purity were performed with a NanoDrop Spectrophotometer (ThermoScientific NanoDrop Technologies). Total protein (30 μg) was mixed with 4X Laemmli sample buffer (Bio-Rad Laboratories, Hercules, CA, USA) and loaded on 4% to 15% Mini-PROTEAN TGX™ Precast Gel (Bio-Rad Laboratories) for electrophoresis. Transfer was done with polyvinylidene fluoride (PVDF) transfer membranes (TRANS-Blot® Turbo™ System, Bio-Rad Laboratories). The membranes were blocked overnight at 4°C followed by primary antibodies—rabbit polyclonal anti-mouse IL-1β (Abcam), rabbit polyclonal anti-mouse IL-6 (Abcam), and rabbit polyclonal anti-mouse GAPDH (Santa Cruz Biotechnology) in blocking buffer overnight at 4°C—and with secondary antibody: horseradish peroxidase-conjugated goat anti-rabbit IgG (Bio-Rad Laboratories) for 1 hour. Immunoblotting bands were visualized by Immun-Star™ WesternC™ Chemiluminescence Kits, and a Gel Doc™ EZ System (Bio-Rad Laboratories) was used for imaging and protein-band assay.

### Microarray

A microarray study of the gene expression profiles of MSCs co-cultured with ECs *in vitro* was previously conducted by our research group and reported recently [12]. From this study, a microarray data set was obtained and processed further. Data analysis was performed by J-Express 2009 software (MolMine, Hafrsfjord, Norway) [27]. The significance analysis of microarrays (SAM) method was used. The data sets were submitted to the Database for Annotation, Visualization, and Integrated Discovery (DAVID) [28] as separate sets of inflammatory related genes, and pathways were determined by Kyoto Encyclopedia of Genes and Genomes (KEGG) pathway mapping. The data are publically available at the National Center for Biotechnology Information with Gene Expression Omnibus accession number GSE63099.

### Statistical analysis

The significance level was set to *P* value of less than 0.05 for all statistical analysis, with n = 6 for each group and time point. SPSS Statistics 21 (IBM, Armonk, NY, USA) was applied for statistical processing and analysis. Two groups (MSC and MSC/EC) were compared with the independent samples *t* test, whereas a multiple comparison one-way analysis of variance was performed to compare three experimental groups (MSC, MSC/EC, and empty scaffold).

## Results

### Gene ontology analysis

**Figure 1:**
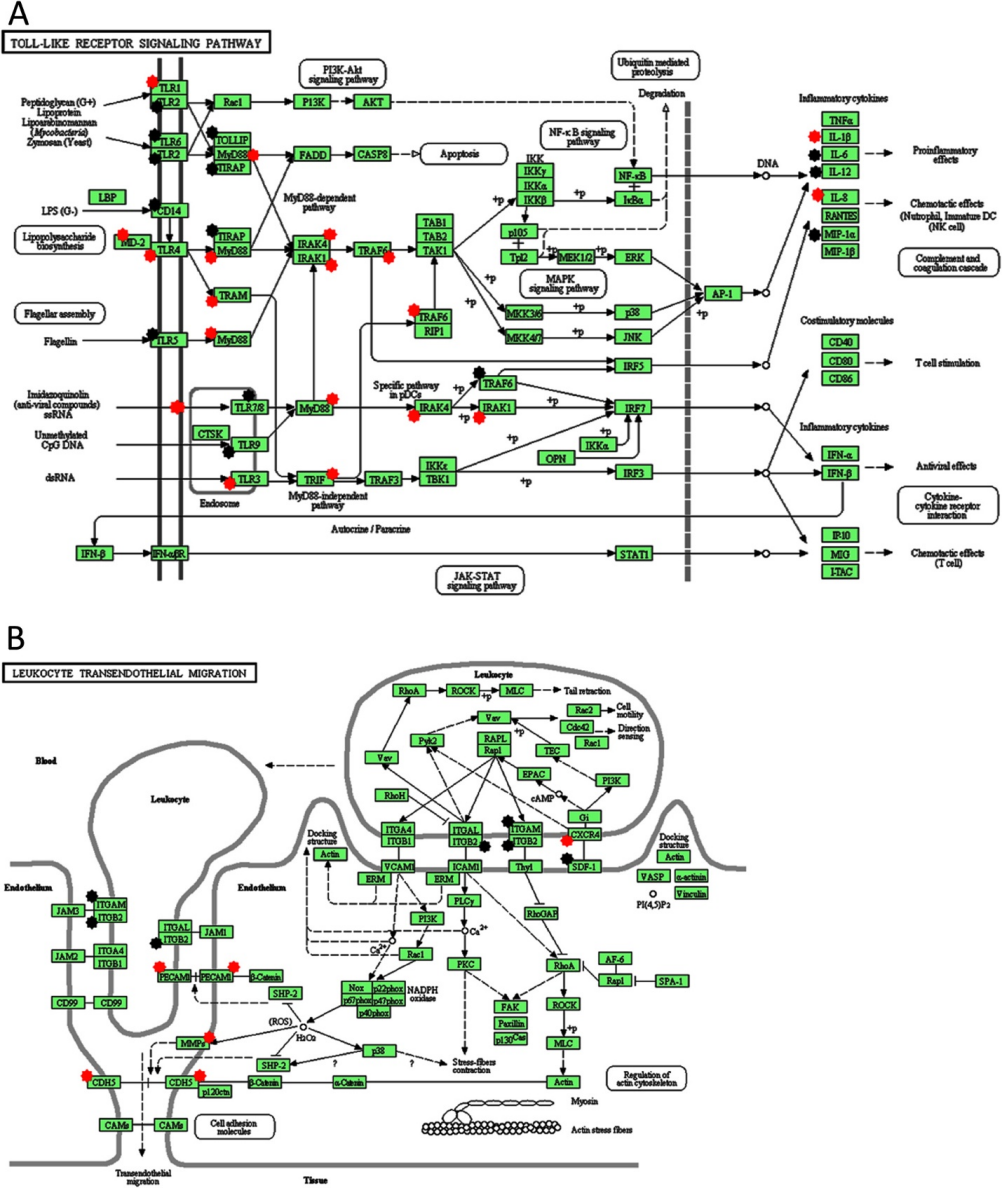
**Genes involved in the leukocyte transendothelial migration and Toll-like receptor signaling pathways.** The over-represented gene lists were submitted to the Database for Annotation, Visualization, and Integrated Discovery (DAVID) [28], and 24 genes were involved in the Toll-like receptor signaling pathway **(A)**, whereas seven genes were involved in the leukocyte transendothelial migration pathway **(B)**. Upregulated genes are labeled with a red star, and downregulated genes are labeled with a black star.

The DAVID pathway database [28] was used to explore and view functionally related genes. Multiple genes in the Toll-like receptor signaling pathway (Figure 1A) and the leukocyte transendothelial migration pathway (Figure 1B) had been influenced by the ECs. The over-represented genes in the respective pathways are presented in Table 2.

**Table 2:** **Up- and downregulated genes from microarray gene ontology analysis comparing MSC (control) and MSC/EC (test)**

PROBE_ID	Gene symbol	Fold change	FDR[i]	PROBE_ID	Gene symbol	Fold change	FDR[i]
**Downregulated**				**Upregulated**			
ILMN_1791447	CXCL12/SDF-1	−5.075	0	ILMN_1686109	CCL23	3.983	0.597
ILMN_1699651	IL6	−4.942	0	ILMN_1740609	CCL15	3.701	0.828
ILMN_1676663	TNFRSF11B	−5.002	0	ILMN_2313672	IL1RL1	9.2	0.224
ILMN_2175912	ITGB2	−2.561	0	ILMN_2184373	IL8	9.954	0
ILMN_1685009	ITGAM	−1.001	77.022	ILMN_1775501	IL1B	1.081	65.3
ILMN_1671353	IL12A	−1.534	10.383	ILMN_2242900	IL1RL1	5.534	0.587
ILMN_1671509	CCL3/ MIP-1α	−1.005	73.358	ILMN_1689518	PECAM1	6.288	0
ILMN_1700353	TRAF6	−1.056	36.202	ILMN_1719236	CDH5	12.865	0
ILMN_1772387	TLR2	−1.024	62.406	ILMN_1801584	CXCR4	1.772	0.67
ILMN_1722981	TLR5	−1.112	11.32	ILMN_1731048	TLR1	1.035	55.07
ILMN_1654560	TLR6	−1.004	75.359	ILMN_2155708	TLR3	1.043	15.787
ILMN_1677827	TLR7	−1.038	33.053	ILMN_1706217	TLR4	1.243	13.508
ILMN_1679798	TLR9	−1.046	44.43	ILMN_1682251	TLR8	1.034	63.84
ILMN_2298366	TLR10	−1.045	57.183	ILMN_1724533	LY96/MD-2	1.006	76.228
ILMN_1740015	CD14	−1.292	11.398	ILMN_1738523	MYD88	1.086	15.187
ILMN_1765523	TOLLIP	−1.078	27.851	ILMN_1651346	TICAM2/TRAM	1.027	70.63
ILMN_1776703	TIRAP	−1.044	10.91	ILMN_2379130	IRAK1	1.138	4.854
ILMN_1724863	TICAM1	−1.069	18.947	ILMN_1692352	IRAK4	1.162	9.159

EC, endothelial cell; FDR[i], false discovery rate; MSC, multipotent stromal cell.

### Cell migration to the construct

**Figure 2:**
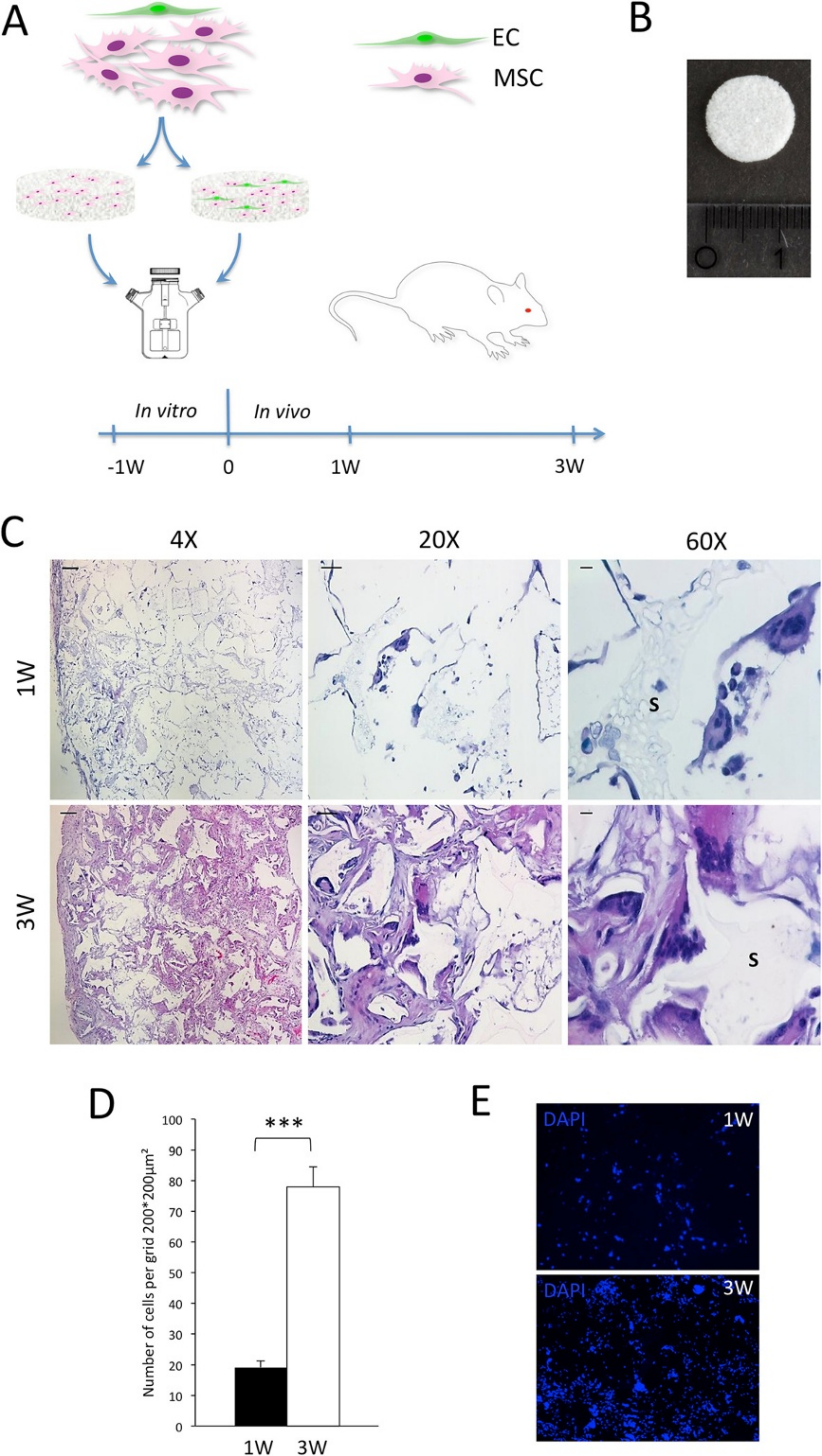
**Implantation of tissue-engineered constructs and subsequent cell migration.** Experimental design of the *in vivo* study is presented in **(A)**, and a macroscopic image of the scaffold used is shown in **(B)**. Stained samples (hematoxylin and eosin) at 1 and 3 weeks **(C)** are shown at 4×, 20×, and 60× magnification with scale bars of 200, 50, and 10 μm, respectively. Multinucleated giant cells on the scaffold surface (S) can be seen at high magnification. **(D)** Quantification of cells migrated into the construct during the experimental period (***P <0.001, n = 6). **(E)** Nuclei of migrated cells visualized with 4′,6-diamidino-2-phenylindole (DAPI) staining. EC, endothelial cell; MSC, multipotent stromal cell.

Hematoxylin-and-eosin staining showed the overall relationship between implanted scaffold and the local cells. One- and three-week samples reflected the recruitment of inflammatory cells in relation to the scaffold and progression over time (Figure 2C). Quantification of DAPI staining was done to show the difference in total cells migrating into the construct during the experimental period, which was significantly higher after 3 weeks compared with 1 week (Figure 2D and 2E) (*P* <0.001). Three representative areas of 200 × 200 μm^2^ per picture, five pictures per section, and five sections per mouse were used for quantification.

### Real-time reverse transcription-polymerase chain reaction

**Figure 3:**
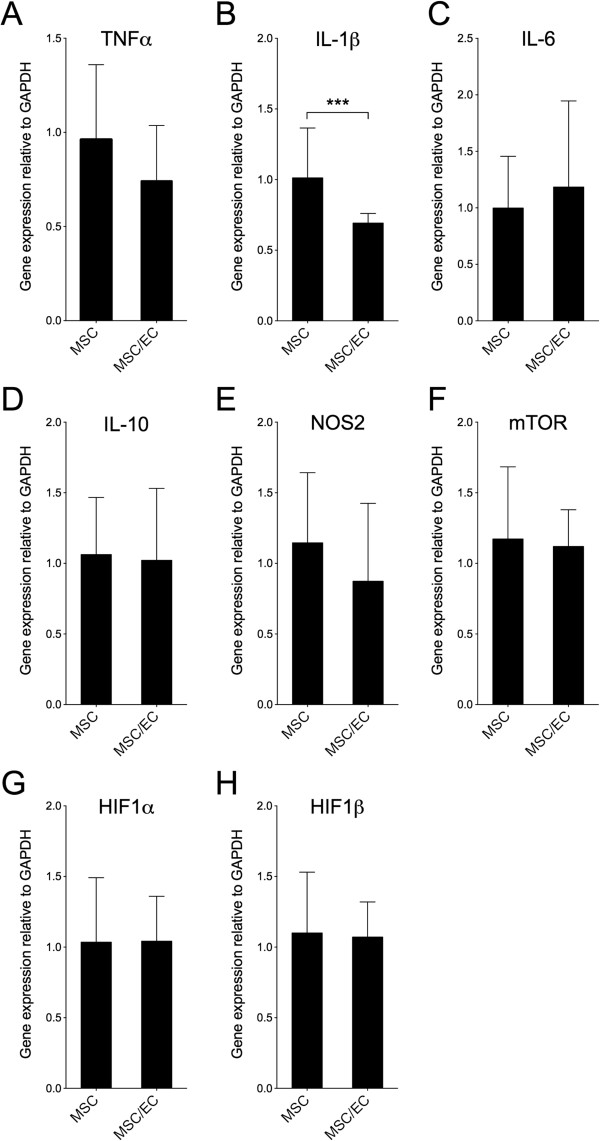
**Real-time reverse transcription-polymerase chain reaction (RT-PCR) for mouse-specific genes after 1 week of implantation** ***in vivo*** **.** Relative gene expression of tumor necrosis factor alpha (TNFα) **(A)**, interleukin-1-beta (IL-1β) **(B)**, IL-6 **(C)**, IL-10 **(D)**, nitric oxide synthase 2 (NOS_2_) **(E)**, mammalian target of rapamycin (mTOR) **(F)**, hypoxia-inducible factor 1 alpha (HIF-1α) **(G)**, and HIF-1β **(H)** comparing multipotent stromal cell (MSC) and MSC/endothelial cell (MSC/EC) constructs. Data are presented as mean ± standard deviation (n = 6). *P <0.05; **P <0.01; ***P <0.001. GAPDH, glyceraldehyde 3-phosphate dehydrogenase.

**Figure 4:**
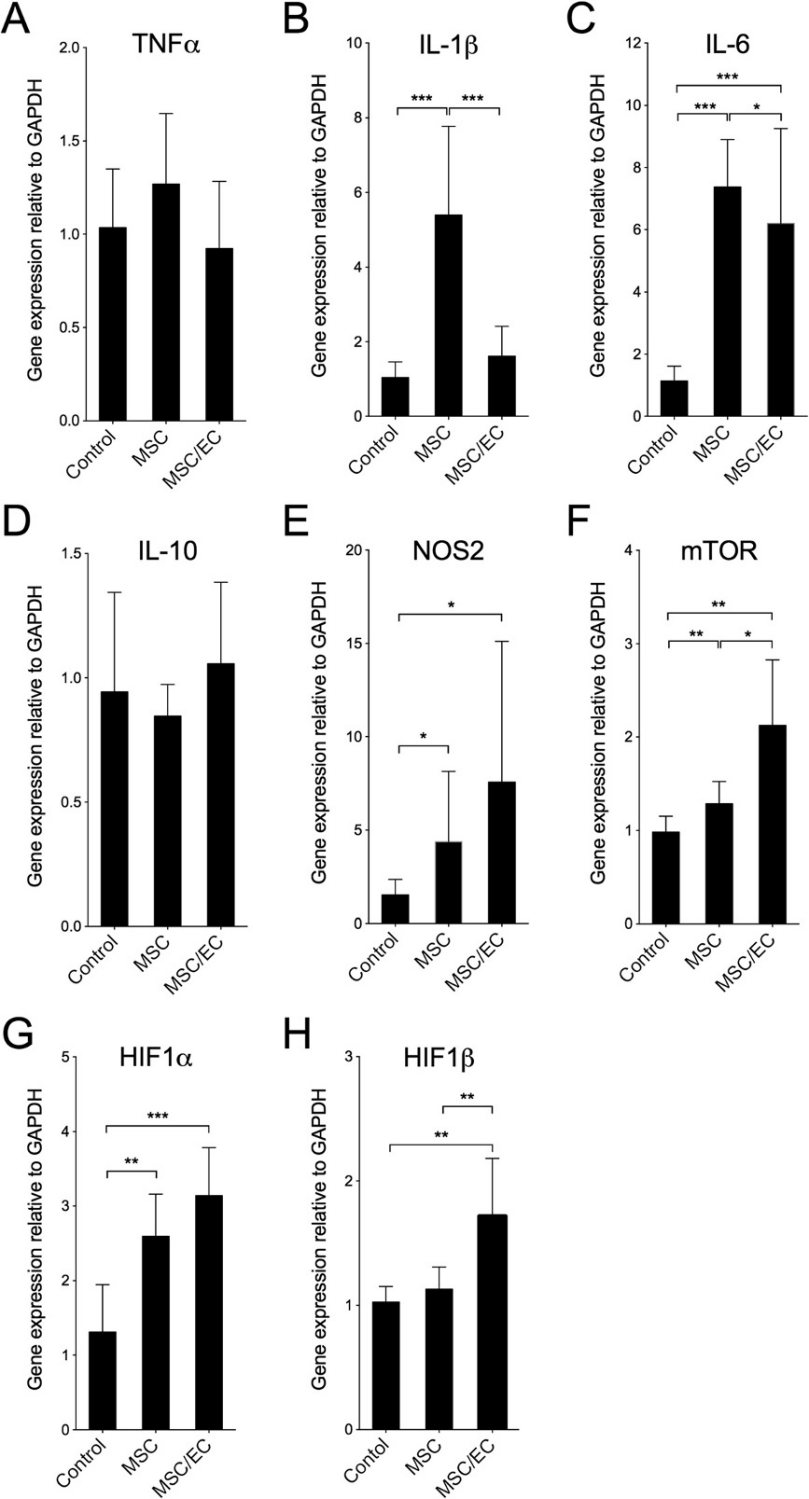
**Real-time reverse transcription-polymerase chain reaction (RT-PCR) for mouse-specific genes after 3 weeks of implantation** ***in vivo*** **.** Relative gene expression of tumor necrosis factor alpha (TNFα) **(A)**, interleukin-1-beta (IL-1β) **(B)**, IL-6 **(C)**, IL-10 **(D)**, nitric oxide synthase 2 (NOS_2_) **(E)**, mammalian target of rapamycin (mTOR) **(F)**, hypoxia-inducible factor 1 alpha (HIF-1α) **(G)**, and HIF-1β **(H)** comparing empty scaffolds with the multipotent stromal cell (MSC) and MSC/endothelial cell (MSC/EC) constructs. Data are presented as mean ± standard deviation (n = 6). *P <0.05; **P <0.01; ***P <0.001. GAPDH, glyceraldehyde 3-phosphate dehydrogenase.

RT-PCR for mouse-specific genes was performed on *in vivo* samples to evaluate the mRNA expression of selected biomarkers important for recruitment of acute and chronic inflammatory cells as well as genes differentially expressed under hypoxic conditions. After 1 week of implantation, most of the target genes evaluated showed no significant differences between the experimental groups. However, IL-1β was significantly downregulated in the MSC/EC group (*P* <0.001) (Figure 3). After 3 weeks of implantation, significant upregulations of pro-inflammatory biomarkers were found, compared with scaffolds implanted without cells. The mRNA expression of IL-1β and IL-6 was higher for the MSC group compared with MSC/EC constructs (Figure 4). There was similar expression of IL-10, an anti-inflammatory marker, for all of the groups. However, the mRNA expression of nitric oxide synthase 2 (NOS_2_) was significantly upregulated in both the MSC and the MSC/EC groups compared with control scaffolds implanted without cells. Hypoxia-inducible factor 1 alpha (HIF-1α), HIF-1β, and mammalian target of rapamycin (mTOR) expression was upregulated in both groups compared with the control. There was also a significant upregulation of these genes in the MSC/EC group compared with the MSC group.

### Immunofluorescent staining and Western blotting

**Figure 5:**
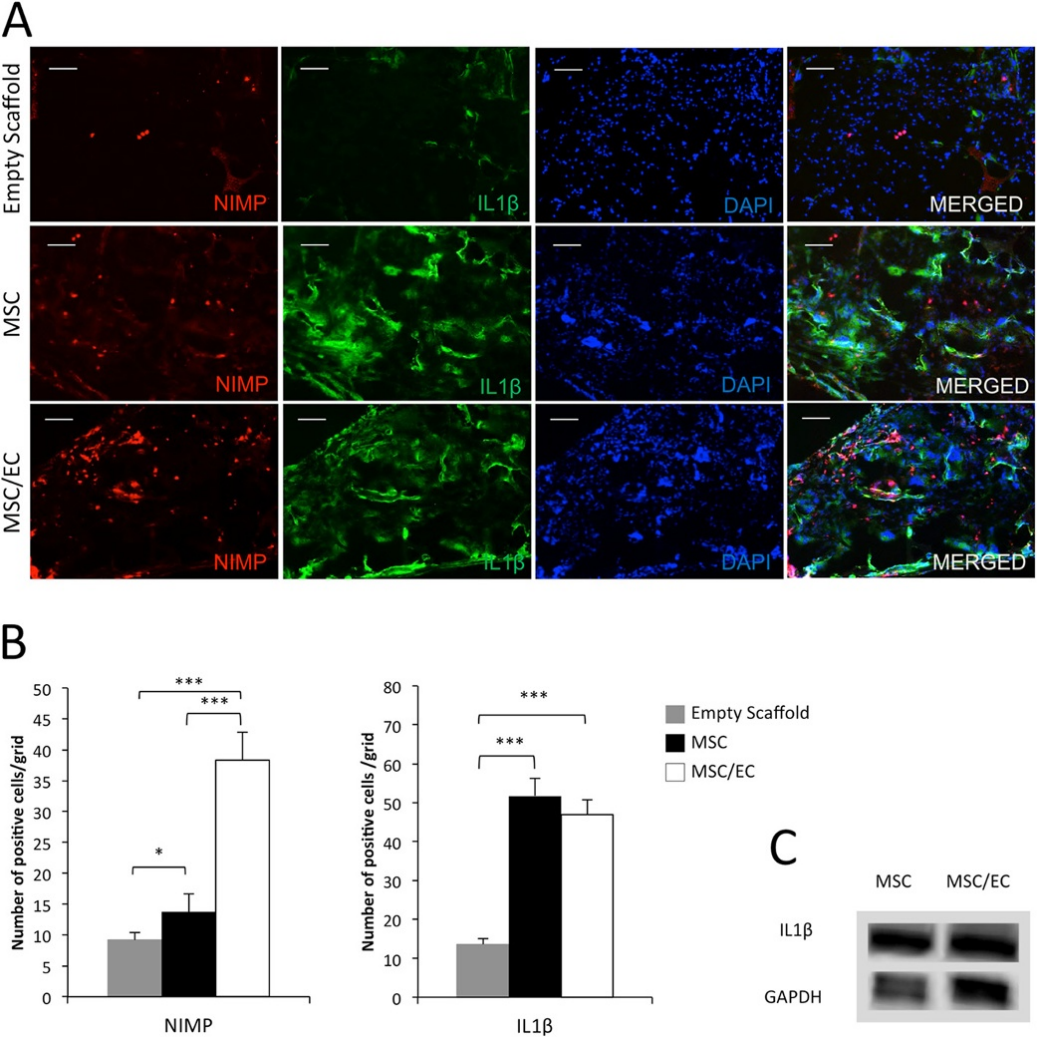
**Transmigration of neutrophils and expression of interleukin-1-beta (IL-1β). (A)** Representative fluorescent micrographs of monoclonal anti-neutrophil antibody (NIMP) and IL-1β staining from the empty scaffold, multipotent stromal cell (MSC), and MSC/endothelial cell (MSC/EC) constructs, using mouse-specific antibodies after 3 weeks of implantation *in vivo* (10× magnification, scale bar = 100 μm). **(B)** The number of neutrophils was significantly higher in the MSC/EC group (***P <0.001, n = 6). The number of IL-1β-positive cells was lower in the MSC/EC group, but this was not a statistically significant difference. Five sections from each mouse and five images for each section were used for the image quantification. **(C)** Relative protein expression of IL-1β in MSC and MSC/EC constructs.

To investigate the association between inflammatory cytokines and migrating leukocytes, we used immunofluorescence double staining on samples retrieved after 3 weeks of implantation (Figure 5A). A remarkable overexpression of neutrophil-positive staining (*P* <0.001) was found in constructs implanted with vascular ECs. IL-1β-positive cells were fewer in the co-culture group than in the mono-culture group, although a statistical difference could not be found (Figure 5B). On the other hand, IL-1β-positive cells were significantly more in the MSC and the MSC/EC groups compared with control scaffolds implanted without cells (*P* <0.001) (Figure 5B).

**Figure 6:**
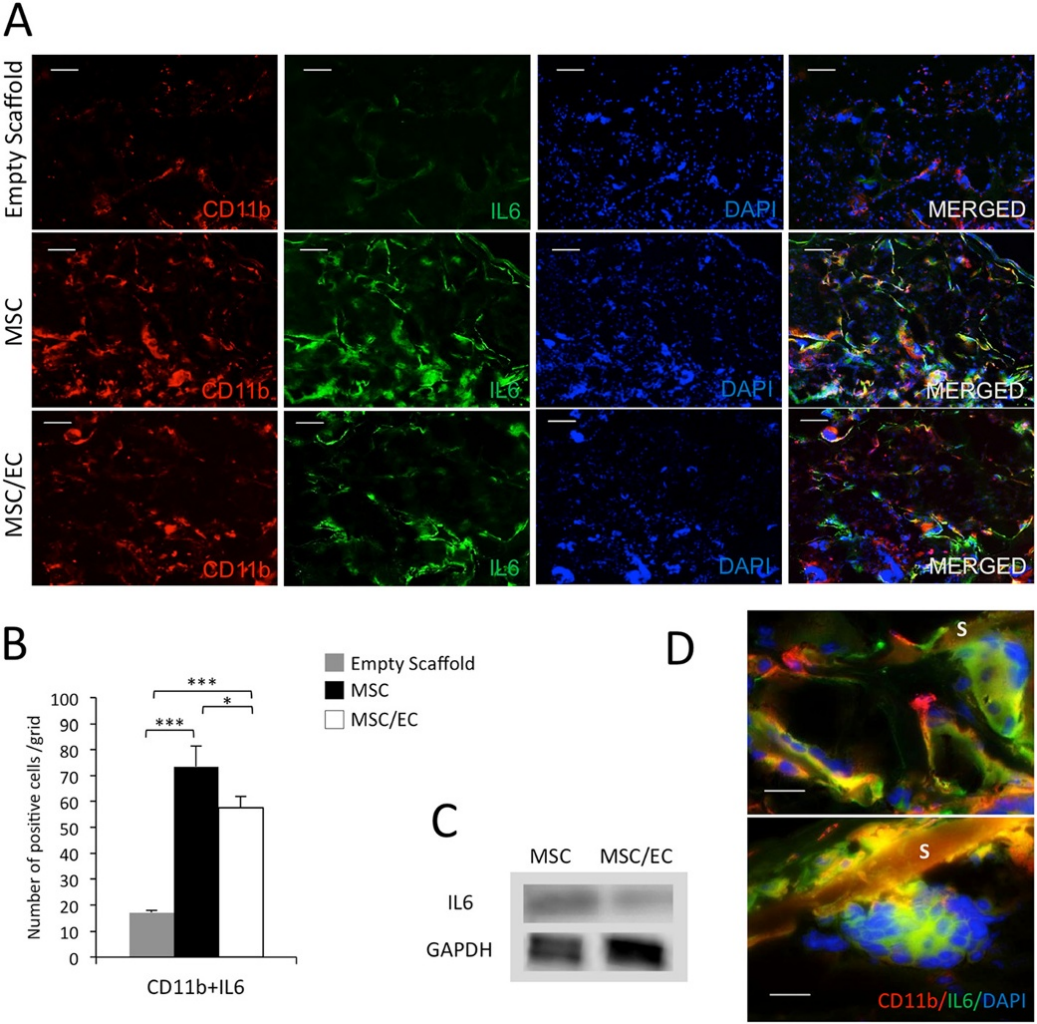
**Density of monocyte-derived cells and expression of interleukin-6 (IL-6). (A)** Representative fluorescent micrographs from the empty scaffold, multipotent stromal cell (MSC), and MSC/endothelial cell (MSC/EC) constructs, showing mouse-specific CD11b- and IL-6-positive multinucleated cells after 3 weeks of implantation *in vivo* (10× magnification, scale bar = 100 μm). Five sections from each mouse and five images for each section were used for the image quantification. **(B)** The number of cells positive for CD11b and IL-6 was lower in the MSC/EC group (*P <0.05, n = 6). **(C)** Relative protein expression of IL-6 in MSC and MSC/EC constructs. **(D)** Giant-cell co-staining with CD11b and IL-6 at 63× magnification. Scale bar = 20 μm. Overall larger giant cells were observed in the MSC/EC group. S, scaffold.

Double staining for IL-6 and CD11b was performed as shown in Figure 5A. IL-6- and CD11b-positive cells were systematically quantified (Figure 6B). There was a significantly lower expression in the tissue-engineered constructs that included ECs compared with mono-culture group. CD11b-positive multinucleated giant cells were observed in close relation to the surface of the scaffolds, contributing to their degradation process (Figure 6B). Giant cells co-stained with CD11b and IL-6 are shown in Figure 5C. IL-6- and CD11b-positive cells were significantly more in the MSC and MSC/EC groups compared with control scaffolds implanted without cells (Figure 6B) (*P* <0.001). Western blotting also revealed that the protein levels of IL-1β (Figure 5C) and IL-6 (Figure 6C) were lower in the co-culture group compared with the mono-culture group.

## Discussion

The aim of this study was to investigate the early inflammatory response to implantation of tissue-engineered constructs containing human cells, and we therefore selected a moderately immunocompromised animal model. A xenograft model with implantation of human MSCs and ECs in NOD/SCID mice was applied. These animals are unable to perform VDJ recombination and subsequent production of antibodies but produce monocyte-derived cells and neutrophils, so that an early innate immune response can be induced [29]. However, analysis of additional inflammatory cells and cytokines associated with the adaptive immune response is one of the limitations of this study. Analysis of the inflammatory response therefore focused on the aforementioned neutrophils and monocyte-derived cells during the first 3 weeks after implantation, and the emphasis was on the transmigrations of these cells, which are events of central importance for creating a favorable microenvironment for tissue regeneration.

Tissue-engineered constructs with or without seeded vascular ECs were evaluated for their effect on transmigration of acute and chronic inflammatory cells following subcutaneous implantation. A two-dimensional culture model *in vitro* was performed to identify pathways for the observed modulation of the inflammatory stimuli, via a microarray gene ontology analysis.

At the site of inflammation, neutrophils are the first line of defense and are later replaced by monocyte-derived cells, T cells, and B cells. The number of neutrophils after 3 weeks of implantation was significantly higher on the constructs co-seeded with MSCs and ECs compared with constructs seeded with MSCs only. In addition, a reduced number of CD11b-positive cells were found for constructs comprising MSCs and ECs. The CD11b-positive cells were observed in close proximity to the surface of the scaffolds, and most cells were large and multinucleated. Biomaterials in tissue engineering are generally considered temporary structural support and delivery devices for bioactive molecules or stem cells or both. The rate of biomaterial degradation is an important event developing in parallel with deposition of new extracellular matrix proteins that are intended to gradually replace the degrading material. The release of degradation products influences the pH in the local environment, potentially prolonging inflammation and influencing tissue repair, and polymer degradation is in part a result of a foreign-body giant-cell reaction [30]. Interestingly, the multinucleated giant cells in the MSC/EC group were generally larger, whereas in the MSC group an increased number of cells were observed, but these were smaller cells with fewer nuclei. These observations were not quantified, and longer observation time would be of further interest to evaluate whether the rate of degradation was influenced by the altered morphology of the multinucleated giant cells. Cross-sectional time points also have limitations in describing the development of acute and chronic inflammation but are useful in observing phase shifts between experimental groups.

Various cytokines are involved in attracting leukocytes from the systemic circulation to the site of a tissue defect. We performed analysis of gene expression on selected inflammatory biomarkers after 1 and 3 weeks of implantation. In the comparison of the MSC and MSC/EC groups, most target genes evaluated after 1 week were not significantly different, except IL-1β, which was clearly downregulated (*P* <0.001) in the MSC/EC group. This downregulation was also found after 3 weeks (*P* <0.001), and IL-1β expression was also strongly upregulated in the MSC group compared with the empty control scaffolds (*P* <0.001). Also, immunostaining and Western blotting for IL-1β showed a downregulation in the MSC/EC group after 3 weeks of implantation, but this was not statistically significant. The expression of IL-1β was, however, inversely related to the number of transmigrated neutrophils detected at the same time point, which was strongly increased with the implantation of ECs. Additional time points could have provided more information about the long-term effect of the release of IL-1β on neutrophil recruitment to the constructs. However, reliable information about the long-term immune response could not be obtained with the animal model selected in this study.

Analysis of human-specific gene and protein expression would also have been of interest to evaluate the inflammatory stimuli delivered directly by the implanted cells. Hematoxylin-and-eosin staining was used to study migration of murine cells into constructs, showing that the scaffolds were not rejected by the host cells. Multiple inflammatory biomarkers were influenced on the mRNA level after 3 weeks of implantation. IL-6 is a cytokine which exhibits both pro- and anti-inflammatory properties, and IL-6 trans-signaling helps to switch from neutrophils to monocytes by activating different chemokines [31]. IL-6 produced by MSCs can inhibit T-cell proliferation [32], and IL-6-dependent prostaglandin E2 (PGE_2_) secretion by MSCs can modulate the immune response by reducing the local inflammation [33]. In addition, MSCs can switch immune profiles from Th1 to Th2 through production of IL-6 [33].

The mRNA expression of IL-6 was significantly upregulated in both the MSC and the MSC/EC groups compared with the empty control scaffolds (*P* <0.001), and the expression in the MSC group was higher than in the MSC/EC group (*P* <0.05). The results were confirmed with immunostaining for IL-6-positive cells and Western blotting.

Overall, the expression of pro-inflammatory cytokines was reduced by adding ECs to the constructs. However, the expression was still higher than in the control group. Previous results have shown that implantations of human cells in this experimental animal model have generated an increased vascular density, and this could be suggested as a consequence of the release of inflammatory cytokines [6].

Cytokines, chemokines, and growth factors are released from a site of injury, attracting native MSCs [34]. Immunomodulatory properties of MSCs are influenced by local recruitment of inflammatory cells which secret pro-inflammatory cytokines interferon-gamma, tumor necrosis factor alpha (TNFα), IL-1α, and IL-1β. These cytokines induce upregulations of inducible nitric oxide synthase (NOS_2_) and chemokines, resulting in accumulation of T cells, B cells, and other immune cells responding to implanted MSCs. Increased levels of nitrous oxide can also suppress immune cell function [35, 36], and the observed upregulated expression of genes encoding NOS_2_ could lead to upregulated expression of NOS_2_ and thus contribute to leukocyte recruitment and function.

Several target genes expressed during tissue hypoxia were upregulated in both experimental groups compared with the control, and the highest expression was detected in the MSC/EC group.

Tissue hypoxia is directly related to inflammation, and inflamed resident and immune cells are highly metabolic and their oxygen consumption is very high. These cells migrate from a high oxygen tension in the blood stream to a hypoxic environment, resulting in production of HIFs. HIFs also exhibit both pro- and anti-inflammatory properties [37, 38] and regulate the microenvironment at the site of inflammation. Cells from the myeloid lineage are also regulated by HIFs, and knockdown of HIF genes results in the impairment of both acute and chronic inflammatory processes [38]. In the present work, upregulated expression of HIF genes could be correlated to an increased acute inflammation. Measurements of tissue oxygen tension would be of interest to further explore these findings.

MSCs express various genes in the Toll-like receptor pathway, recruiting cells to sites of injury by activating both the innate and adaptive immune system. TLR4 and TLR3 and their ligands have the capability to induce nuclear factor-κβ activity as well as the production of IL-6, IL-8, and CXCL10 [39]. It has been postulated that MSCs could also be attracted to a site by similar mechanisms. MSCs express different Toll-like receptors which directly affect the cell homing of other MSCs. TLR4-primed MSCs regulate pro-inflammatory cytokines, whereas TLR3-primed MSCs regulate anti-inflammatory cytokines. The production of IL-6 and IL-8 is a downstream mechanism of Toll-like receptors [40, 41]. Our gene ontology data detected a total of 24 over-represented genes in the Toll-like receptor pathway after co-culture with ECs, modulating the production of inflammatory cytokines, and with both TLR3 and TLR4 upregulated in the MSC/EC group. The pro-inflammatory cytokine IL-6 was downregulated by fivefold, whereas IL-8—a cytokine acting as both a potent chemoattractant and an inducer of neutrophil activation—was upregulated by 10-fold in the MSC/EC group [42]. The CXCL12/CXCR4 axis is a key factor for homing neutrophils within the bone marrow and blood and has a major role in leukocyte trafficking in both homeostasis and inflammation [43]. The CXCL12/CXCR4 axis was downregulated in the MSC/EC group, and the influence of ECs on Toll-like receptor signaling and CXCL12/CXCR4 axis signaling presents possible pathways for activation of leukocyte transendothelial migration.

## Conclusions

The addition of vascular ECs to tissue-engineered constructs clearly influenced transmigration of leukocytes involved in both acute and chronic inflammation. After 3 weeks of implantation, the number of neutrophils was significantly increased, whereas the number of monocyte-derived cells was decreased, suggesting a phase shift in the inflammatory response with the presence of ECs. Evaluation of gene and protein expression showed altered expression of inflammatory cytokines, and gene ontology analysis revealed multiple genes in the leukocyte transmigration and Toll-like receptor pathways regulated by ECs.

## Authors’ information

SB-B and TOP are shared first authors.

## Abbreviations

DAPI: 4′,6-diamidino-2-phenylindole
DAVID: Database for Annotation, Visualization, and Integrated Discovery
EC: endothelial cell
GAPDH: glyceraldehyde 3-phosphate dehydrogenase
HIF: hypoxia-inducible factor
IL: interleukin
MSC: multipotent stromal cell
NIMP: monoclonal anti-neutrophil antibody
NOD/SCID: non-obese diabetic/severe combined immunodeficient
NOS_2_: nitric oxide synthase 2
poly(LLA-co-DXO): poly(L-lactide-co-1,5-dioxepan-2-one)
RT-PCR: reverse transcription-polymerase chain reaction.
